# anamiR: integrated analysis of MicroRNA and gene expression profiling

**DOI:** 10.1186/s12859-019-2870-x

**Published:** 2019-05-14

**Authors:** Ti-Tai Wang, Chien-Yueh Lee, Liang-Chuan Lai, Mong-Hsun Tsai, Tzu-Pin Lu, Eric Y. Chuang

**Affiliations:** 1 Graduate Institute of Biomedical Electronics and Bioinformatics, National Taiwan University, Taipei, 10617 Taiwan; 2 Graduate Institute of Physiology, National Taiwan University, Taipei, 10051 Taiwan; 3 Bioinformatics and Biostatistics Core, Center of Genomic Medicine, National Taiwan University, Taipei, 10055 Taiwan; 4 Institute of Biotechnology, National Taiwan University, Taipei, 10672 Taiwan; 5 Center for Biotechnology, National Taiwan University, Taipei, 10672 Taiwan; 6 Institute of Epidemiology and Preventive Medicine, National Taiwan University, Taipei, 10055 Taiwan

**Keywords:** MicroRNA, Gene expression, Functional enrichment analysis, R/Bioconductor

## Abstract

**Background:**

With advancements in high-throughput technologies, the cost of obtaining expression profiles of both mRNA and microRNA in the same individual has substantially decreased. Integrated analysis of these profiles can help to elucidate the functional effects of RNA expression in complex diseases, such as cancer. However, fundamental discrepancies are observed in the results from microRNA-mRNA target gene prediction algorithms, and few packages can be used to analyze microRNA and mRNA expression levels simultaneously.

**Results:**

To address these issues, an R package, anamiR, was developed. A total of 10 experimental/prediction databases were integrated. Two analytical functions are provided in anamiR, including the single marker test and functional gene set enrichment analysis, and several parameters can be changed by users. Here we demonstrate the potential application of the anamiR package to 2 publicly available microarray datasets.

**Conclusion:**

The anamiR package is effective for an integrated analysis of both RNA and microRNA profiles. By characterizing biological functions and signaling pathways, this package helps identify dysregulated genes/miRNAs from biological and medical experiments. The source code and manual of the anamiR package are freely available at https://bioconductor.org/packages/release/bioc/html/anamiR.html.

**Electronic supplementary material:**

The online version of this article (10.1186/s12859-019-2870-x) contains supplementary material, which is available to authorized users.

## Background

With advancements in high-throughput technologies, the cost of analyzing multiple gene expression profiles in the same individual has dropped substantially. Many studies have described attempts to analyze RNA and microRNA (miRNA) profiles simultaneously. To date, only a few miRNAs and their target genes have been validated through biological experiments [1], and thus prediction algorithms have been widely used to identify potential miRNA-gene interaction pairs. The most popular method for predicting target genes of miRNAs is that of matching the 3’UTR of an mRNA to the “seed region” (a conserved sequence of 2 to 8 nucleotides) of an miRNA, which assumes perfect Watson-Crick complementarity between the mRNA 3’UTR and the miRNA. In addition, the seed region can be used to classify families and species of miRNAs. Consequently, the seed region has been demonstrated to be a key element of miRNA-target gene prediction [2]. However, other research has shown that the pairing mechanism between miRNA and mRNA can occur anywhere along the entire mRNA, which suggests that algorithms focusing on the seed regions can only identify a subset of all potential miRNA-target gene pairs [3]. To address this issue, other popular computational approaches, such as free energy minimization and machine learning, have been developed. Measuring the minimum free energy can help to assess the stability of binding sites between miRNA and target genes. A predicted miRNA-target pair with lower free energy indicates that the binding is more stable and thus more likely to be a true result [2, 4]. With the rapid accumulation of massive amounts of data, machine learning algorithms have also been implemented in many prediction algorithms through training processes containing the entire dataset of known miRNA targets [4]. In such approaches, important features that can facilitate identification of possible target genes from miRNAs can be revealed. Good performance has been reported for support vector machine [5] and generalized linear model [6] algorithms. Furthermore, two machine learning based algorithms were implemented to dissect the associations between miRNAs and diseases [7, 8], and their results demonstrated that utilizing data mining approaches may effectively improve the prediction accuracy. However, prediction results from different algorithms usually show large discrepancies [9]. For example, the proportion of the miRNA-gene pairs that can be predicted by all 8 algorithms in our system is only 0.001% (Additional file 1: Table S1). Therefore, inconsistent prediction results still pose a major challenge to advanced analyses of miRNA targets. To address this issue, one possible solution is to use several algorithms to analyze several experimental datasets at the same time [10]. In this study, we developed an R package, anamiR, for analyzing miRNA expression and gene expression concurrently. A total of 8 target prediction algorithms and 2 experimentally validated miRNA databases were included. Users can analyze genome-wide expression profiles of both miRNA and RNA without applying a preset filter. The anamiR package provides 2 major functions: the single marker test and functional gene set enrichment analysis. The former can be used to identify differentially expressed RNAs/miRNAs, and the latter is used to characterize their biological functions and signaling pathways. Alternatively, users can pre-select gene sets and/or pathways of interest, and the anamiR package can reveal dysregulated genes/miRNAs involved in them. The anamiR package can substantially reduce the time and effort required to perform an integrated analysis of genome-wide miRNA and gene expression.

## Implementation

### Characteristics of anamiR

**Fig. 1:**
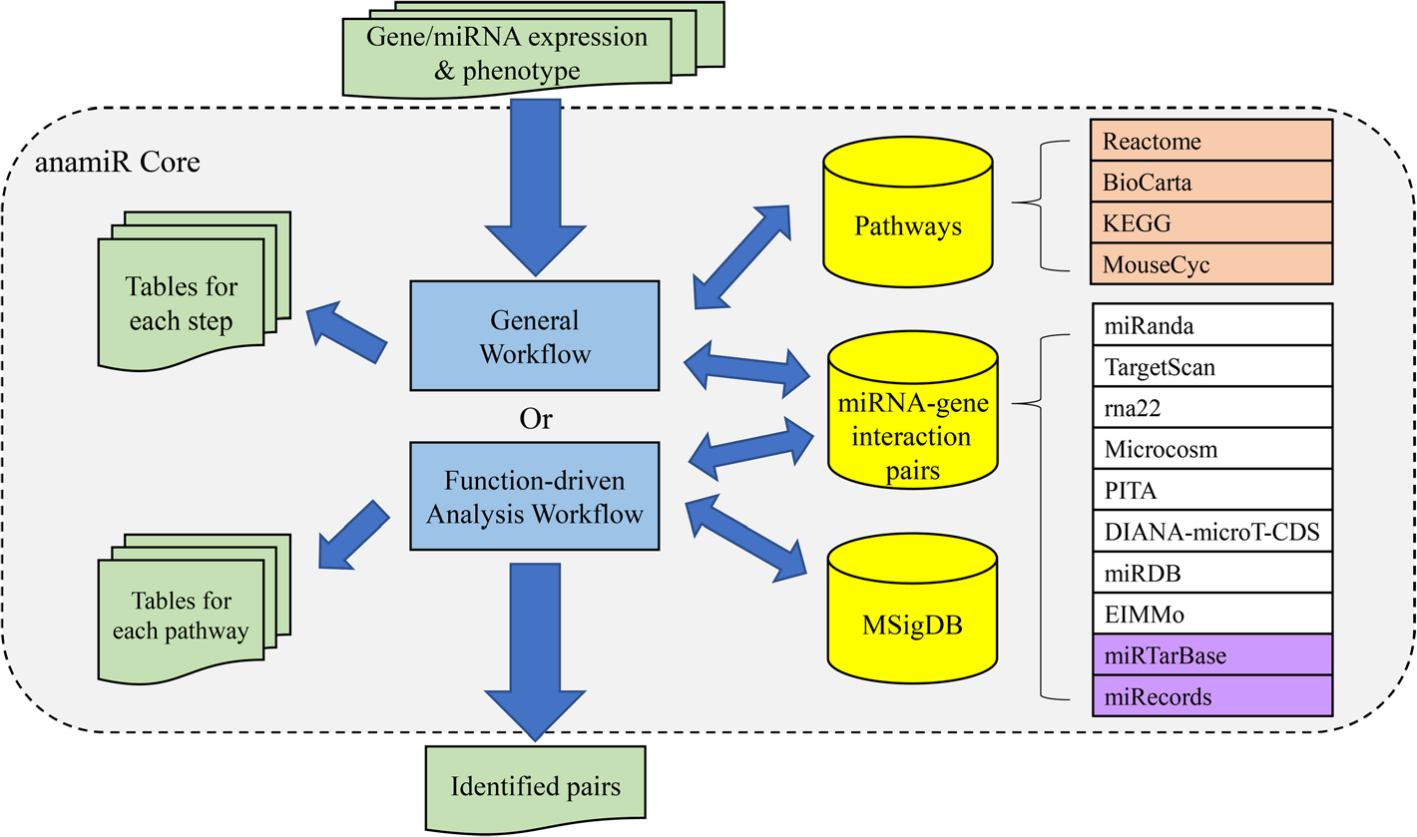
An overview of anamiR. The rounded rectangle with a dotted outline shown in grey indicates the core of the anamiR package. The input data and output results are shown in green. The blue boxes represent two major workflows in the package. Three databases including pathway information from four datasets (orange boxes), collections of miRNA- gene interaction pairs from eight prediction programs (white boxes), and two experimentally validated datasets (purple boxes), and MSigDB are shown in yellow cylinders

The overall structure of the anamiR package is illustrated in Fig. 1. We collected both predicted and validated datasets containing the potential target genes of miRNAs, as well as biological functions and pathway information, in the anamiR database. To address the issue of low consistency across independent prediction algorithms, we utilized an approach that selects the intersection of the prediction results. The same approach has been used in our previous study [10]. Compared with the previous study, we have added 8 new or updated prediction algorithms, including different in silico approaches for predicting potential miRNA-gene pairs, such as strong base pairing between 3′ UTR of mRNAs and seed regions with variously complementary types (i.e., TargetScan [11] and EIMMo [12]), additionally considering thermal dynamic stability of binding sites (i.e., rna22 [13], miRanda [14], MicroCosm [15], and PITA [16]), incorporating machine learning methods (i.e., DIANA-microT-CDS [17] and miRDB [5]), and 2 experimentally validated datasets (i.e., miRecords [18], and miRTarBase [19]) in anamiR. The numbers of miRNA-gene interaction pairs are summarized in Additional file 1: Table S2. Also, four datasets containing information on biological functions and pathways in both humans and mice were embedded in anamiR, including KEGG [20], Reactome [21], BioCarta, and MouseCyc [22]. Currently, only one published R package, miRComb [23], has similar function to anamiR, and comparisons of the two are shown in Additional file 1: Table S3. The primary advantages of anamiR with respect to miRComb are the inclusion of experimentally validated datasets and the greater number of functional prediction algorithms.

### Analytical workflows in the anamiR package

**Fig. 2:**
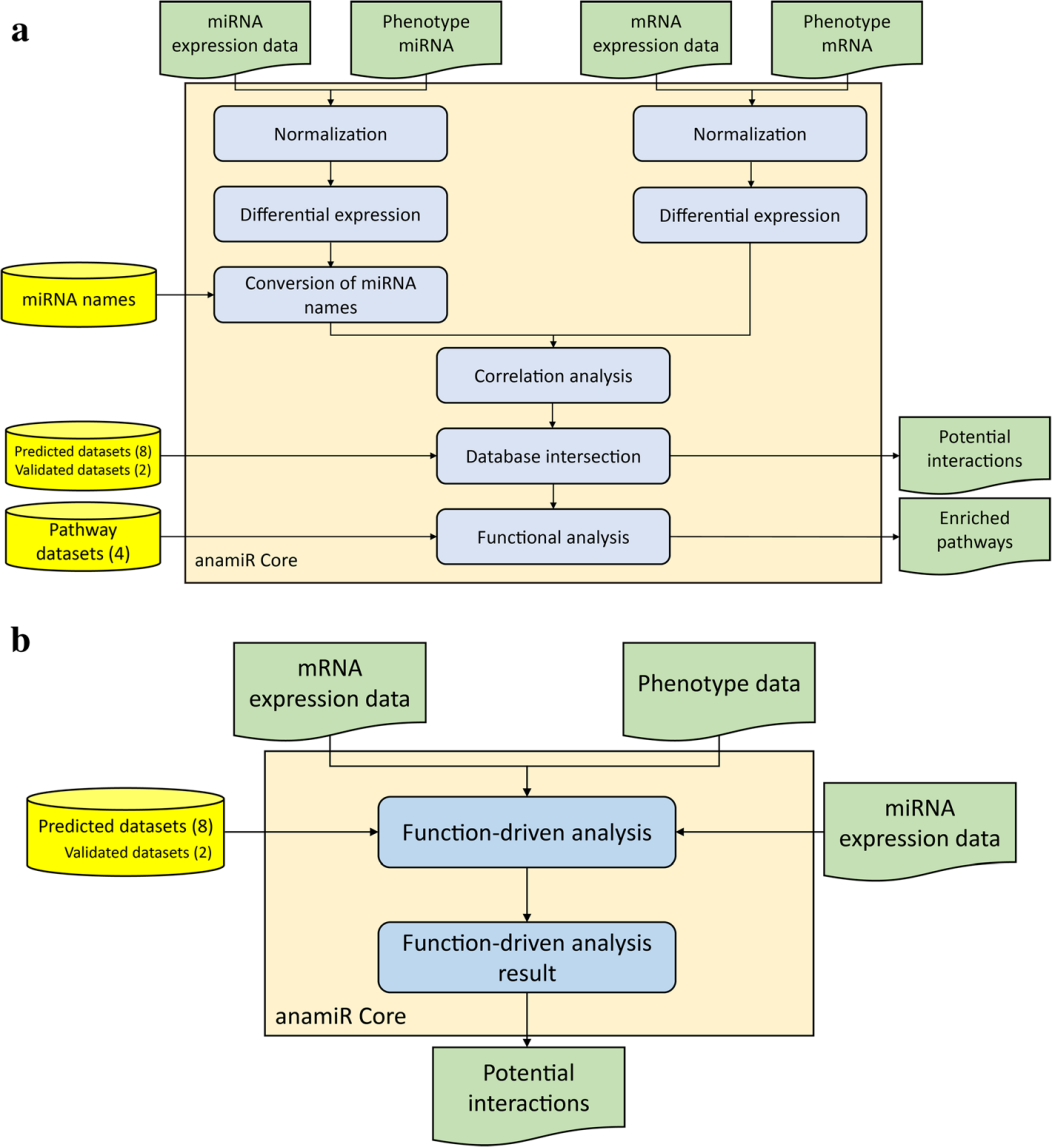
The proposed workflows in the anamiR package. (**a**) The general workflow provides six steps including normalization (optional), differential expression analysis, conversion of miRNA names (optional), correlation analysis, database intersection, and functional analysis. miRNA-gene interaction pairs and correspondingly enriched pathways can be identified from the workflow using both mRNA and miRNA expression data. (**b**) The function-driven analysis workflow is performed to identify significantly dysregulated pathways and to obtain potential miRNA-gene interaction pairs using genome-wide expression profiles. Each box shown in blue represents a function in the anamiR package. The input data and output results are shown in green. Yellow cylinders indicate databases for query and numbers of dataset collections are shown in parentheses

In the anamiR package, we have provided a general workflow that contains several statistical tests for performing differential expression analysis, correlation analysis, and functional analysis (Fig. 2a). Users need to provide 2 data sheets of genome-wide expression levels of both miRNA and genes, and information on sample grouping is also required to perform the statistical analyses. For each statistical test and analysis step, all results can be generated as output, and most analysis parameters are flexible and can be changed by the user. To estimate the chances of randomly identifying a significantly enriched biological function, a permutation test is performed.

In addition to the general workflow, the anamiR package provides another workflow called function-driven analysis (Fig. 2b). Users can select the biological functions/pathways of interest on which to perform these integrated analyses. All genes in the corresponding functional pathways are analyzed, with no filtering based on their statistical *P*-values. Such an approach can not only reduce the number of tests required to obtain an answer, but also take moderate gene expression changes into consideration, even if they do not reach statistical significance.

### Statistical approaches

In the anamiR package, we have provided four statistical tests and/or algorithms by which to identify differentially expressed genes and/or miRNAs: limma [24], t-test, Wilcoxon rank sum test, and DESeq2 [25]. The default *P*-value threshold for the statistical tests is defined as 0.05 and the default threshold for the expression ratio between two phenotypes is set at 0.5. Both parameters are user-adjustable. The Benjamini-Hochberg method is performed to address the multiple testing issue. Three measurements of the trend in the expression levels between gene and miRNA are provided: Pearson correlation coefficient, Kendall rank correlation coefficient, and Spearman’s rank correlation coefficient. Since the expression level of a miRNA is negatively correlated to the expression level of its target gene, the default cutoff of the correlation (also user-adjustable) is defined as − 0.5, which corresponds to moderate correlation (Additional file 1: Table S4 and Table S5). For those genes showing negative correlation with their regulatory miRNA, a hypergeometric test is performed to identify significantly enriched pathways. Lastly, to address the possibility of identifying a significant pathway through miRNA enrichment analysis by random chance [26], a permutation test is embedded in the anamiR package. The random chance of identifying a significant pathway is obtained by randomly selecting the same number of genes in the enriched pathway and calculating the enriched *P*-value. This procedure is repeated 5000 times to generate a null distribution, and the empirical *P*-value is determined by comparing the enrichment *P*-value obtained from the real data with the *P*-value from the null distribution. For the function-driven analysis workflow, we employed the gage package [27] to perform genome-wide functional enrichment analysis, and users can manually set the number of functional terms of interest.

## Results and discussion

### Dataset description

To demonstrate the potential applications of the anamiR package, 2 examples from real microarray datasets are provided below. The first example (GSE16558) has 60 patients with multiple myeloma and 5 normal controls [28], whereas the second example (GSE60371) contains 48 prostate cancer patients and 6 normal controls [29]. Both datasets are composed of paired miRNA and mRNA expression data.

### Example 1: multiple myeloma (GSE16558)

We analyzed the GSE16558 dataset by the general workflow algorithm (Fig. 2a), using limma with the default parameters shown in the supplementary information (Additional file 1: Table S6). The 5 interaction pairs with the highest correlation coefficients between miRNA and gene are shown in Table 1. A previous study has identified miR-485-5p as having dysregulated expression levels in multiple myeloma [30], and its target gene, *JMY*, is reported as a mediating and regulatory protein of *TP53* [31]. Intriguingly, 3 out of the top 5 enriched pathways were related to *TP53* (Table 2), suggesting the interaction between miR-485-5p and *JMY* deserves further investigation in multiple myeloma patients.

**Table 1:** Top 5 miRNA-gene interaction pairs with negative correlation coefficients (GSE16558)

miRNA	Target gene	Number of prediction algorithms	Experimentally validated	Correlation	References^a^
hsa-miR-485-5p	*JMY*	3		−0.522	[30, 32]
hsa-miR-622	*IRF2BP2*	3		−0.469	[33, 34]
hsa-miR-186-5p	*SNF8*	3		−0.438	[34]
hsa-let-7b-5p	*RHOG*	1	V	−0.435	[35]
hsa-miR-155-5p	*PELI1*	4	V	−0.433	[36]

^a^The miRNA and/or gene was reported to be related to multiple myeloma

**Table 2:** Pathways identified by functional enrichment analysis (GSE16558)

Category	Function	Number of genes	Number of genes targeted by miRNAs	*P*-value^a^	Empirical *p*-value^b^
Reactome	Gene expression	1588	15	6.06E-07	0.0002
Reactome	Transcriptional regulation by TP53	345	7	6.90E-06	0.0002
KEGG	FoxO signaling pathway	126	4	0.0001	0.0002
BioCarta	p53 signaling pathway	13	2	0.0003	0.0004
BioCarta	Hypoxia and p53 in the cardiovascular system	21	2	0.0008	0.0005

^a^The *P*-value was obtained by the hypergeometric test

^b^The *P*-value was obtained by the permutation test

Alternatively, the top 5 enriched pathways from the function-driven analysis (Fig. 2b) are shown in Table 3. Notably, the most significant pathway was related to ribosome function, and 3 of the 5 interaction pairs with the largest negative correlations include miR-485-5p, in agreement with the results from the general workflow (Additional file 1: Table S7). Therefore, these results demonstrate that anamiR is able to identify important interaction pairs of miRNA and target genes in a specific disease.

**Table 3:** Top 5 pathways identified by function-driven analysis (GSE16558)

Category	Function	Number of genes	Number of genes targeted by miRNAs	Number of interaction pairs with correlation coefficient ≤ − 0.3
KEGG	Ribosome	88	29	62
Reactome	Peptide Chain Elongation	153	31	65
Reactome	3 UTR mediated translational regulation	176	39	79
Reactome	Influenza Viral RNA Transcription and Replication	169	36	78
Reactome	Nonsense Mediated Decay Enhanced by The Exon Junction Complex	176	41	78

### Example 2: prostate cancer (GSE60371)

Similar to the previous example, we utilized the general workflow algorithm with the default parameters in GSE60371 (Additional file 1: Table S6). Notably, 2 miRNAs from the miR-320 family were identified (Table 4), and their down-regulation has been reported in prostate cancer [37]. Intriguingly, the overexpression of *UBE2C* was reported in prostate cancer [38] and its miRNA regulator is miR-320d. Taken together, the dysregulation of the miR-320 family and *UBE2C* deserve further investigation in prostate cancer.

**Table 4:** Top 5 miRNA-gene interaction pairs with negative correlation coefficients (GSE60371)

miRNA	Gene	Number of predicted algorithms	Correlation	References^a^
hsa-miR-1260a	*SH3BP5*	3	−0.789	
hsa-miR-320d	*UBE2C*	3	−0.721	[39]
hsa-miR-320d	*BICD1*	3	−0.709	[40]
hsa-miR-320b	*BICD1*	3	− 0.706	[41]
hsa-miR-1260a	*LMNA*	3	− 0.705	[42]

As shown in Tables 5 and 6, the results showed that the proportion of miRNA-gene pairs showing negative Pearson correlation coefficients increase along with the number of analyzed algorithms, suggesting better prediction performances can be achieved by the integration of multiple algorithms.

**Table 5:** The proportion of miRNA-gene pairs with negative correlations (GSE16558)

Pearson Correlation	Number(s) of algorithm		
	=0 algorithm(s) (*N* = 19,114 pairs)	> = 1 algorithm(s) (*N* = 5009 pairs)	> = 3 algorithm(s) (*N* = 1174 pairs)
Correlation < − 0.1	196 (1.03%)	125 (3.91%)	52 (4.43%)
Correlation < − 0.3	29 (0.15%)	17 (0.34%)	9 (0.77%)
Correlation < −0.5	0 (0.0%)	0 (0.00%)	0 (0.00%)

**Table 6:** The proportion of miRNA-gene pairs with negative correlations (GSE60371)

Pearson Correlation	Number(s) of algorithm		
	=0 algorithm(s) (*N* = 74,968 pairs)	> = 1 algorithm(s) (*N* = 29,798 pairs)	> = 3 algorithm(s) (*N* = 6611 pairs)
Correlation < −0.1	533 (0.71%)	450 (1.51%)	233 (3.52%)
Correlation < −0.3	290 (0.39%)	238 (0.79%)	133 (2.01%)
Correlation < −0.5	55 (0.07%)	43 (0.14%)	19 (0.29%)

## Conclusions

The anamiR package provides an integrated approach for identifying paired mRNA and miRNA expression profiles. The general workflow is utilized to predict the target genes and their associated functional pathways for miRNA simultaneously. Within gene sets and pathways of interest, the function-driven analysis workflow is applied to identify miRNA-gene interaction pairs from among the significant gene sets and pathways. We believe that approaches considering the associations between mRNAs and miRNAs, as well as regulation of genes and pathways, can provide insight into dysfunction in cancers.

## Availability and requirements

- **Project name:** anamiR
- **Project home page:** https://bioconductor.org/packages/release/bioc/html/anamiR.html
- **Operating system(s):** Platform independent
- **Programming language:** R
- **Other requirements:** R (> = 3.3.3), SummarizedExperiment (> = 1.1.6), Bioconductor (> = 3.4), stats, DBI, limma, lumi, agricolae, RMySQL, DESeq2, SummarizedExperiment, gplots, gage, S4Vectors
- **License:** GNU GPLv2
- **Any restrictions to use by non-academics:** None

## Additional file

Additional file 1 **Table S1.** The total potential number of miRNA-gene pairs obtained by tallying different prediction algorithms. **Table S2.** Number of miRNA/gene interaction pairs in the prediction algorithms and experimentally validated databases included in the anamiR package. **Table S3.** Characteristics of anamiR and miRComb. **Table S4.** Pairs with negative correlation coefficients (GSE16558). **Table S5.** Pairs with negative correlation coefficients (GSE60371). **Table S6.** The default parameters used in the examples. **Table S7.** Top 5 interaction pairs with negative correlation coefficients in the 5 pathways identified by function-driven analysis. (DOCX 38 kb)

## Abbreviations

KEGG: Kyoto Encyclopedia of Genes and Genomes
miRNA: microRNA
