# Supplementary material for: anamiR: integrated analysis of MicroRNA and gene expression profiling
Source: BMC Bioinformatics. 2019 May 14;20:239. doi: 10.1186/s12859-019-2870-x (PMC6518761; doi:10.1186/s12859-019-2870-x)
Supplement: Supplementary file 1 — Table S1. The total potential number of miRNA-gene pairs obtained by tallying different prediction algorithms. Table S2. Number of miRNA/gene interaction pairs in the prediction algorithms and experimentally validated databases included in the anamiR package. Table S3. Characteristics of anamiR and miRComb. Table S4. Pairs with negative correlation coefficients (GSE16558). Table S5. Pairs with negative correlation coefficients (GSE60371). Table S6. The default parameters used in the examples. Table S7. Top 5 interaction pairs with negative correlation coefficients in the 5 pathways identified by function-driven analysis. (DOCX 38 kb) [file 12859_2019_2870_MOESM1_ESM.docx]

Supplementary Data

## Supplementary Tables

**Table S1** The total potential number of miRNA-gene pairs obtained by tallying different prediction algorithms

| **Number of algorithms** | **Potential pairs (%)** | **Cumulative percentage of pairs** |
| --- | --- | --- |
| 8 | 192 (0.001%) | 0.001% |
| 7 | 4,314 (0.033%) | 0.034% |
| 6 | 30,652 (0.234%) | 0.268% |
| 5 | 122,003 (0.934%) | 1.202% |
| 4 | 382,110 (2.928%) | 4.130% |
| 3 | 1,046,425 (8.019%) | 12.149% |
| 2 | 2,516,091 (19.281%) | 31.430% |
| 1 | 8,948,698 (68.570%) | 100.000% |
| Total | 13,050,485 (100.0%) | -- |

| **Table S2** Number of miRNA/gene interaction pairs in the prediction algorithms and experimentally validated databases included in the anamiR package | | | |
| --- | --- | --- | --- |
| **Database** | **Number of pairs (human)** | **Number of pairs (mouse)** | **Reference** |
| DIANA-microT-CDS | 5161315 | 2324223 | [1] |
| miRanda | 2894164 | 1737143 | [2] |
| miRDB | 947783 | 623791 | [3] |
| rna22 | 2907774 | 928222 | [4] |
| TargetScan | 447142 | 442499 | [5] |
| Microcosm | 700571 | 550387 | [6] |
| PITA | 204953 | 143196 | [7] |
| EIMMo | 6381664 | 1239484 | [8] |
| miRecords | 1502 | 367 | [9] |
| miRTarBase | 321744 | 39201 | [10] |

| **Table S3** Characteristics of anamiR and miRComb | | |
| --- | --- | --- |
| **Characteristics** | **anamiR** | **miRComb** |
| **Databases** |  |  |
| Experimentally validated datasets | **✓** |  |
| Prediction algorithms (number) | **✓ (8)** | **✓ (2)** |
| **Statistical analyses** |  |  |
| Differential expression analysis | **✓** | **✓** |
| Correlation analysis | **✓** | **✓** |
| Functional analysis | **✓** | **✓** |
| **Function-driven analysis** | **✓** |  |
| **miRNA ID conversion** | **✓** | **✓** |
| **Available in Bioconductor** | **✓** |  |

Table S4 Pairs with negative correlation coefficients (GSE16558)

| **Correlation coefficient** | **Potential pairs (%)** | **Cumulative percentage of pairs** |
| --- | --- | --- |
| -0.7 | 0 (0.0%) | 0.0% |
| -0.5 | 13 (0.06%) | 0.06% |
| -0.3 | 1,775 (9.29%) | 9.35% |
| 0 | 17,326 (90.65%) | 100.0% |
| Total | 19,114 (100.0%) | -- |

Table S5 Pairs with negative correlation coefficients (GSE60371)

| Correlation coefficient | Potential pairs (%) | Cumulative percentage of pairs |
| --- | --- | --- |
| -0.7 | 124 (0.17%) | 0.17% |
| -0.5 | 4,797 (6.40%) | 6.57% |
| -0.3 | 27,380 (36.52%) | 43.09% |
| 0 | 42,667 (56.91%) | 100.0% |
| Total | 74,968 (100.0%) | -- |

| **Table S6** The default parameters used in the examples | | |
| --- | --- | --- |
| **General Workflow** | | |
| Method | Function | Parameters |
| Differential Expression | differExp_discrete() | method = limma  p_value.cutoff = 0.05  logratio = 0.5  p_adjust.method = “BH” |
| Correlation | negative_cor() | cut.off = -0.3/-0.7(this depends) |
| Databases Intersection | database_support() | Sum.cutoff = 3 |
| Enrichment Analysis | enrichment() | per_time = 5000 |
| **Function-Driven Analysis** | | |
| Method | Function | Parameters |
| Function Driven | GSEA_ana() | pathway_num = 10 |
| Analyzing Report | GSEA_res() | DE_method = “limma”  cor_cut = 0 |

| **Table S7** Top 5 interaction pairs with negative correlation coefficients in the 5 pathways identified by function-driven analysis | | | |
| --- | --- | --- | --- |
| **miRNA** | **Gene** | **Number of predicted algorithms** | **Correlation** |
| hsa-miR-485-5p | *RPS23* | 2 | -0.410 |
| hsa-miR-223-3p | *RPL38* | 2 | -0.398 |
| hsa-miR-485-5p | *RPS9* | 1 | -0.383 |
| hsa-miR-223-3p | *RPL23A* | 2 | -0.382 |
| hsa-miR-485-5p | *RPL29* | 1 | -0.432 |

## References

1. Paraskevopoulou MD, Georgakilas G, Kostoulas N, Vlachos IS, Vergoulis T, Reczko M, Filippidis C, Dalamagas T, Hatzigeorgiou AG: **DIANA-microT web server v5.0: service integration into miRNA functional analysis workflows**. *Nucleic acids research* 2013, **41**(Web Server issue):W169-173.

2. Betel D, Wilson M, Gabow A, Marks DS, Sander C: **The microRNA.org resource: targets and expression**. *Nucleic acids research* 2008, **36**(Database issue):D149-153.

3. Wang X: **miRDB: a microRNA target prediction and functional annotation database with a wiki interface**. *RNA (New York, NY)* 2008, **14**(6):1012-1017.

4. Loher P, Rigoutsos I: **Interactive exploration of RNA22 microRNA target predictions**. *Bioinformatics (Oxford, England)* 2012, **28**(24):3322-3323.

5. Agarwal V, Bell GW, Nam JW, Bartel DP: **Predicting effective microRNA target sites in mammalian mRNAs**. *Elife* 2015, **4**.

6. Griffiths-Jones S, Saini HK, van Dongen S, Enright AJ: **miRBase: tools for microRNA genomics**. *Nucleic acids research* 2008, **36**(Database issue):D154-158.

7. Kertesz M, Iovino N, Unnerstall U, Gaul U, Segal E: **The role of site accessibility in microRNA target recognition**. *Nat Genet* 2007, **39**(10):1278-1284.

8. Gaidatzis D, van Nimwegen E, Hausser J, Zavolan M: **Inference of miRNA targets using evolutionary conservation and pathway analysis**. *BMC Bioinformatics* 2007, **8**:69.

9. Xiao F, Zuo Z, Cai G, Kang S, Gao X, Li T: **miRecords: an integrated resource for microRNA-target interactions**. *Nucleic acids research* 2009, **37**(Database issue):D105-110.

10. Chou CH, Chang NW, Shrestha S, Hsu SD, Lin YL, Lee WH, Yang CD, Hong HC, Wei TY, Tu SJ *et al*: **miRTarBase 2016: updates to the experimentally validated miRNA-target interactions database**. *Nucleic acids research* 2016, **44**(D1):D239-247.
